# Breast cancer cell secretome analysis to decipher miRNA regulating the tumor microenvironment and discover potential biomarkers

**DOI:** 10.1016/j.heliyon.2023.e15421

**Published:** 2023-04-14

**Authors:** Riley Feser, Reid M. Opperman, Braydon Nault, Sujit Maiti, Vincent C. Chen, Mousumi Majumder

**Affiliations:** a Department of Biology, Brandon University, 3rd Floor, John R. Brodie Science Centre, 270 18th Street, Brandon, MB, Canada, R7A 6A9; b Department of Chemistry, Brandon University, 4th Floor, John R. Brodie Science Centre, 270 18th Street, Brandon, MB, Canada, R7A 6A9

**Keywords:** Breast cancer, MicroRNA, miR526b, miR655, Secretome, Biomarker, Tumor microenvironment (TME), Estrogen receptor-positive (ER+), Luminal A, Bioinformatics, Gene expression

## Abstract

MicroRNA (miRNA/miR) 526 b- and miR655-overexpressed tumor cell-free secretions regulate the breast cancer tumor microenvironment (TME) by promoting tumor-associated angiogenesis, oxidative stress, and hypoxic responses. Additionally, premature miRNA (pri-miR526b and pri-miR655) are established breast cancer blood biomarkers. However, the mechanisms of how these miRNAs regulate the TME has yet to be investigated. Mass spectrometry analysis of miRNA-overexpressed cell lines MCF7-miR526b, MCF7-miR655, and miRNA-low MCF7-Mock cell-free secretomes identified 34 differentially expressed proteins coded by eight genes. In both miRNA-high cell secretomes, four markers are upregulated: *YWHAB*, *SFN*, *TXNDC12*, and *MYL6B,* and four are downregulated: *PEA15*, *PRDX4*, *PSMB6*, and *FN1*. All upregulated marker transcripts are significantly high in both total cellular RNA pool and cell-free secretions of miRNA-high cell lines, validated with quantitative RT-PCR. Bioinformatics tools were used to investigate these markers' roles in breast cancer. These markers' top gene ontology functions are related to apoptosis, oxidative stress, membrane transport, and motility supporting oncogenic miR526b- and miR655-induced functions. Gene transcription factor analysis tools were used to show how these miRNAs regulate the expression of each secretory marker. Data extracted from the Human Protein Atlas showed that YWHAB, SFN, and TXNDC12 expression could distinguish early and late-stage breast cancer in various breast cancer subtypes and are associated with poor patient survival. Additionally, immunohistochemistry analysis showed the expression of each marker in breast tumors. A stronger correlation between miRNA clusters and upregulated secretory markers gene expression was found in the luminal A tumor subtype. YWHAB, SFN, and MYL6B are upregulated in breast cancer patient's blood, showing biomarker potential. Of these identified novel miRNA secretory markers, SFN and YWHAB successfully passed all validations and are the best candidates to further investigate their roles in miRNA associated TME regulation. Also, these markers show the potential to serve as blood-based breast cancer biomarkers, especially for luminal-A subtypes.

## 1 Introduction

In 2021, breast cancer surpassed lung cancer as the most diagnosed cancer worldwide, accounting for 11.7% of all cancer cases [1]. In addition, breast cancer-related fatalities continue to rise globally, with a 9.3% increase from 2018 to 2020 [1,2]. Early detection can improve breast cancer patient survival rate to 99% [3]. Mammograms are the most effective, affordable, and highly sensitive breast cancer screening procedure used globally. However, routine mammogram screening begins at age 50 in most countries [4]. With the incidence of breast cancer in younger populations increasing, finding accessible, minimally invasive, and sensitive early detection biomarkers is a global need. Blood-based biomarkers can serve as sensitive and specific detection tools to diagnose breast cancer in the early stages.

The complexity of breast cancer has led to the development of specialized treatment regimens that are dependent on tumor stage, grade, and the presence or absence of hormone receptors (HR; estrogen receptor (ER) and progesterone receptor (PR) and human epidermal growth factor receptor 2 (HER2)). The majority of diagnosed breast tumors (68%) are subtype luminal A (HR+/HER2-), followed by 10% that are triple-negative (HR-/HER2-), 10% are luminal B (HR+/HER2+), and 4% are HER2-enriched (HR-/HER2+) [5]. Improvement in patient survival is possible with precision medication and personalized therapy [6].

miRNAs are short non-coding RNAs that regulate post-transcriptional gene expression. In breast cancer, miRNA dysregulation is associated with cancer progression and metastasis [7]. The overexpression of cyclooxygenase-2 (COX-2), an inflammatory enzyme, in poorly metastatic luminal A breast cancer cell line MCF7 upregulated two oncogenic miRNAs, miR526b and miR655 [8,9]. Overexpression of miR526b and miR655 in luminal A breast tumor cell line MCF7 and HER2 positive SKBR3 promoted aggressive breast cancer phenotypes such as epithelial to mesenchymal transition (EMT), cell migration, invasion, induction of cancer stem cells (CSCs), tumor-associated angiogenesis, and lymphangiogenesis, oxidative stress, and hypoxia [[8], [9], [10], [11], [12]].

During oncogenic transformation, the tumor cell secretes various growth factors, ligands, and metabolites, to regulate diverse autocrine and paracrine regulation, which alters the tumor microenvironment (TME) to promote metastasis [7,13]. The secretome is the collection of proteins secreted by cells into extracellular space. It makes up a substantial amount (13–20%) of the human proteome and plays essential roles in cell migration, cell signaling, and cell-cell communication [14]. miRNA-overexpressed cell secretions and metabolites induced oxidative stress, tumor-associated angiogenesis, and lymphangiogenesis and enhanced hypoxic responses [[10], [11], [12]]. However, the compositions of miR526b and miR655 secretomes are unknown. Additionally, secretory proteins found in bodily fluids can be detected in blood and are potential biomarkers [14,15]. Both miR526b and miR655 expressions in human breast tumors are associated with poor breast cancer patient survival [8,9]. Premature miRNAs, pri-miR526b and pri-miR655, can be detected in blood plasma, showing a signature of highly sensitive breast cancer biomarkers, and pri-miR526b can distinguish stage one tumor compared to benign samples; hence it is an early diagnostic breast cancer biomarker [16]. Here, the secretomes of miRNA-overexpressed cell lines were systematically analyzed to identify novel secretory markers regulating the TME in breast cancer and to investigate their biomarker potential.

## 2 Results

### 2.1 Identification of proteins differentially expressed in MCF7-miR526b and MCF7-miR655 cell secretomes

**Fig. 1:**
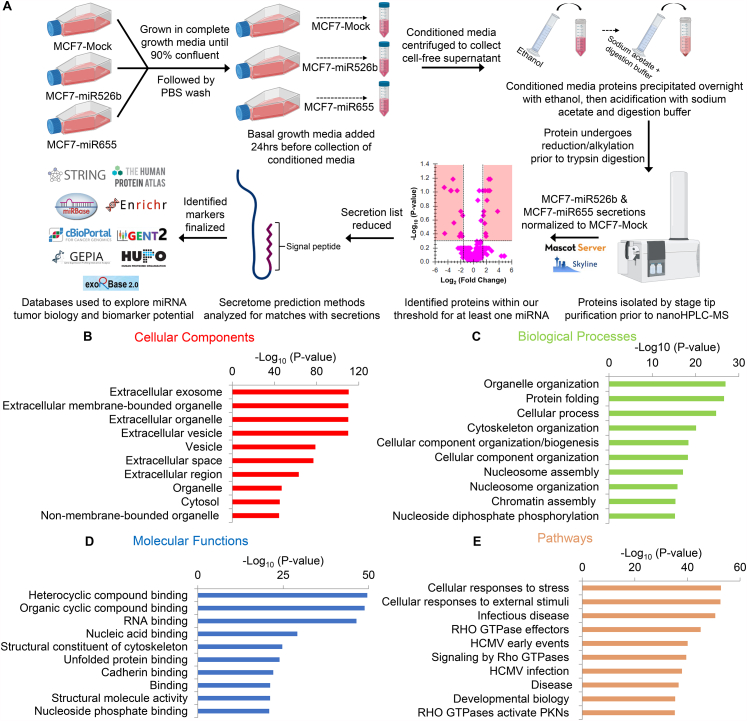
Schematic overview of the project and the top ten most enriched GOs and pathways. (**A**) Summary of the miRNA-high secretome project. Top ten most significant (**B**) cellular components, (**C**) biological processes, (**D**) molecular functions, and (**E**) pathways of differentially expressed secretory molecules in MCF7-miR526b and MCF7-miR655 secretomes shown in -log_10_ p-value.

A systematic analysis of MCF7-miR526b, MCF7-miR655, and MCF7-Mock cell secretomes (Fig. 1A) with Nano-high-performance liquid chromatography-mass spectrometry (nanoHPLC-MS) determined a total of 1535 secreted proteins, coded by 442 genes, differentially expressed in both MCF7-miRNA-high cell secretomes (Supplementary Data 1). A gene ontology (GO) analysis of differentially secreted proteins was completed to identify statistically significant GO enrichment functions. The identified 1535 proteins are involved in 426 biological processes, 145 molecular functions, and 458 pathways, which can be found across 168 different cellular components, with 58.1% of the proteins mapped to the extracellular region (Supplementary Data 2). The seven most significant cellular components found are all extracellular associated, including extracellular exosome (*p* = 3.34 × 10^−111^), extracellular membrane-bounded organelle (*p* = 6.95 × 10^−111^), extracellular organelle (*p* = 9.27 × 10^−111^), extracellular vesicle (*p* = 1.15 × 10^−110^), vesicle (*p* = 1.3 × 10^−79^), extracellular space (*p* = 1.05 × 10^−77^), and extracellular region (*p* = 8.37 × 10^−64^) (Fig. 1B). This indicates that most identified differentially secreted proteins are major regulators of the TME.

The most significant biological process, organelle organization (*p* = 1.19 × 10^−27^), allows proper organization of the cytoskeleton, Golgi apparatus, vacuoles, and extracellular vesicles (Fig. 1C). Another important biological process includes cellular processes (*p* = 1.79 × 10^−25^), such as cellular export, cell adhesion, cell-cell fusion, and cell communication. These processes are crucial to ensure proper protein secretion, transportation, and contribute to cell-cell communication. Eight of the top 10 most significant molecular functions involved the binding of molecules such as heterocyclic compounds (*p* = 2.41 × 10^−50^), organic cyclic compounds (*p* = 1.48 × 10^−49^), RNA (*p* = 3.55 × 10^−47^), nucleic acid (*p* = 7.42 × 10^−30^), unfolded protein (*p* = 1.19 × 10^−24^), cadherin (*p* = 8.38 × 10^−23^), and nucleoside phosphate (*P* = 1.24 × 10^−21^) (Fig. 1D). This indicates that secretory proteins have major roles in macro-molecule biogenesis and transportation. The most significantly enriched pathway is cellular response to stress (*p* = 1.78 × 10^−53^) (Fig. 1E), which directly relates to the reported roles of miR526b and miR655 in promoting oxidative stress in breast cancer [10]. The next enriched pathway is cellular response to external stimuli (*p* = 2.96 × 10^−53^). This result supports our earlier report that miRNA-high cells secrete angiogenesis and lymphangiogenesis stimulating factors that enhance the migration and tube formation abilities of HUVECs [11].

### 2.2 Data curation and threshold determination

**Fig. 2:**
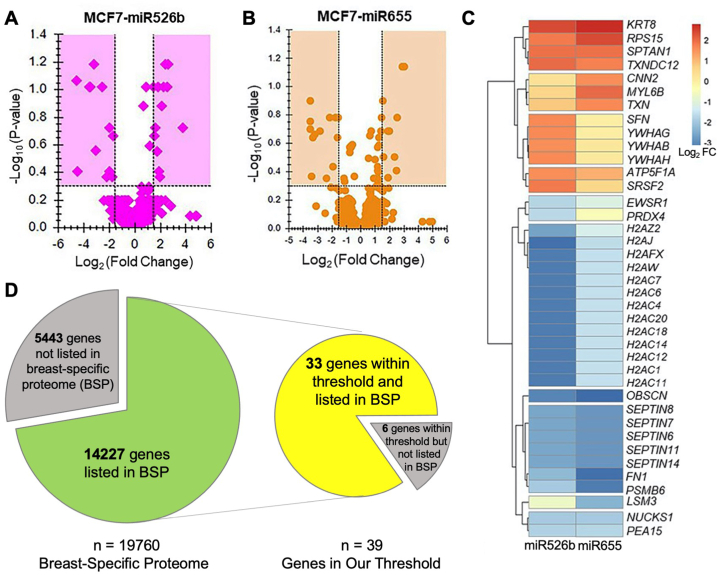
Total secretory markers identified in the MCF7-miR526b and MCF7-miR655 secretome and threshold determination. Volcano plot highlighting proteins within the threshold in (**A**) MCF7-miR526b and (**B**) MCF7-miR655 secretomes. Highlighted regions of the volcano plot show protein IDs within the threshold. (**C**) Heatmap showing log_2_ FC values of the total protein-coding genes found from MCF7-miR526b and MCF7-655 volcano plots. Red shows increased expression, and blue shows decreased expression. (**D**) Comparison of identified secretome genes in the breast-specific proteome (BSP). Green pie chart indicates all human protein-coding genes that are listed in the BSP. Yellow pie chart shows which protein-coding genes from this study overlap with the BSP list. Gray pieces in C and D indicate listed markers not matched or found in our list.

To identify the most important differentially expressed secretory proteins, a >1.5/<-1.5 log_2_ fold change (FC) and >0.3 -log_10_ p-value threshold was established. This threshold determined 96 proteins coded by 32 genes in the MCF7-miR526b secretome (Fig. 2A) and 95 proteins coded by 29 genes in the MCF7-miR655 secretome (Fig. 2B). In both miRNA secretomes, a total of 136 proteins coded by 39 genes (Fig. 2C) were identified. Of the 39 protein-coding genes, 13 are upregulated, and 26 are downregulated in both miRNA-secretomes (Fig. 2C). Interestingly, many protein-coding genes were from the same protein family, including 13 from the H2A histone family, five from Septin, four from 14-3-3, and three from thioredoxin (Figure S1A). Most protein families are grouped on the agglomerative hierarchical clustering heatmap, including histone H2A, Septin, and 14-3-3 (Fig. 2C). This is further confirmed by these protein families' protein-protein interaction network, as they are also grouped together (Figure S1B).

Next, differential secretory proteins were analyzed to ensure they could be found in the breast-specific proteome (BSP). There are 19,670 human protein-coding genes, of which 14,227 are listed in the BSP (Fig. 2D). From the identified 39 genes in this study, 33 were found in the BSP. The six genes absent in the BSP (*H2AC1*, *H2AC12, H2AC14, H2AC18*, *OBSCN*, and *SEPT14*) were excluded from further analysis.

### 2.3 Validation of differential secretory proteins using secretome prediction methods

**Fig. 3:**
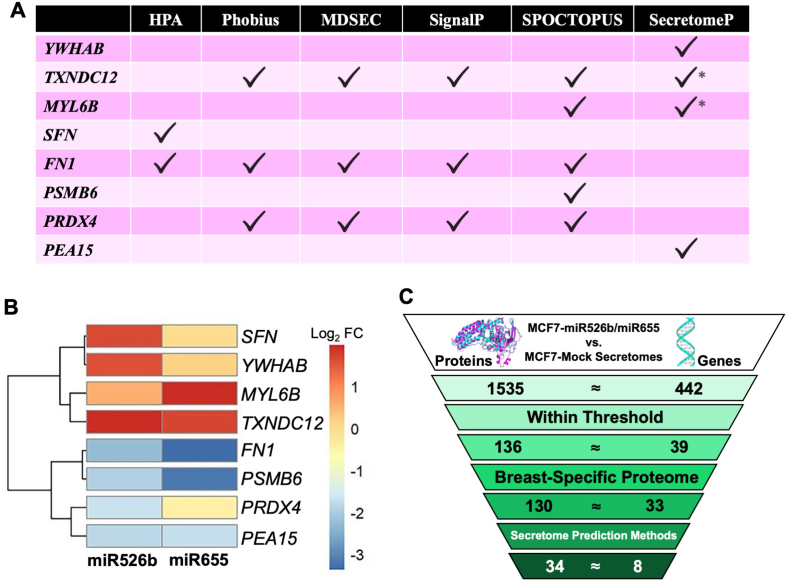
Genes identified by secretome prediction methods and overview of data curation pipeline. (**A**) Overview of each protein-coding gene and validation method used for secretome prediction method. * = Secretome markers were considered classically secreted if found in both classical and non-classical secretion methods. (**B**) Heatmap showing the log_2_ FC of the eight identified markers in miR526b and miR655 which passed the threshold. (**C**) Each step of the data curation process highlights how the final list of secretome markers was achieved.

The 33 genes within the BSP were analyzed by five classical (signal peptide) secretome prediction methods (HPA, Majority Decision-Based Method for Secreted proteins (MDSEC), Phobius, SignalP, and SPOCTOPUS), and one non-classical (no signal peptide) secretome prediction method, SecretomeP. This process determines which of the differentially expressed secretory proteins in miRNA-high cell secretomes are known secretory proteins. Six protein-coding genes, Thioredoxin family containing 12 (*TXNDC12*), Myosin light chain 6 B (*MYL6B*), Stratifin (*SFN*), Fibronectin 1 (*FN1*), Proteasome 20 S subunit beta 6 (*PSMB6*), and Peroxiredoxin 4 (*PRDX4*), follow the classical secretory pathway. Two protein-coding genes, Tyrosine 3-monooxygenase/tryptophan 5-monooxygenase activation protein beta (*YWHAB*) and proliferation and apoptosis adaptor protein 15 (*PEA15*), follow the non-classical secretory pathway (Fig. 3A, Supplementary Data 3). Secretome markers were considered classically secreted if found in both classical and non-classical secretion methods [17]. Of the eight identified secretome markers, four are upregulated (*YWHAB, TXNDC12*, *MYL6B*, and *SFN*), and four are downregulated (*FN1*, *PSMB6*, *PRDX4,* and *PEA15*) by miR526b or miR655 (Fig. 3B).

Overall, our data curation pipeline identified eight secretome markers from the beginning 1535 secreted proteins, as summarized in Fig. 3C. These eight secretome markers present in both miRNA-secretomes will be further investigated through bioinformatic approaches and validated via mRNA gene expression, examining their relationships with miR526b and miR655 and evaluating their blood-based biomarker potential.

### 2.4 Gene ontology analysis of eight identified secretome markers

**Fig. 4:**
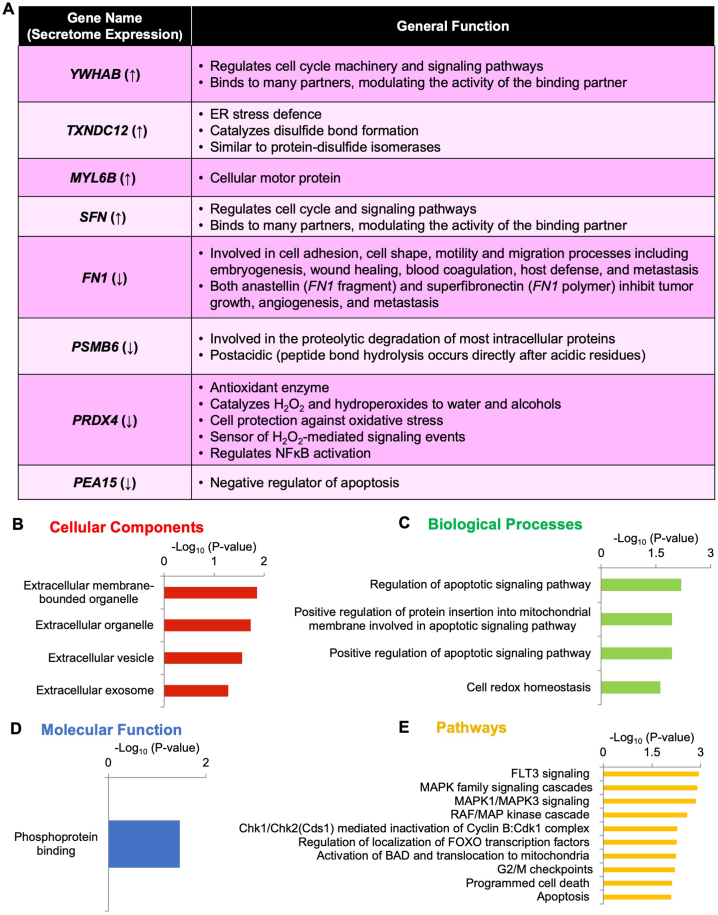
Eight secretome markers GO analysis. (**A**) List of secretome marker's general functions. Shared significantly enriched (**B**) cellular components, (**C**) biological processes, (**D**) molecular component GOs, and (**E**) pathways regulated by the secretome markers shown in −log_10_ (p-value).

First, the general functions and individual cellular components, biological processes, and molecular functions of each secretome marker were determined using GO analysis (Fig. 4A and Table S1). Interestingly, many secretome markers’ functions and GO enrichments overlapped with miR526b- and miR655-induced breast cancer phenotypes, such as induction of oxidative stress as reported earlier [10]. For example, identified *PRDX4* and *TXNDC12* are associated with oxidative stress response. *TXNDC12* is upregulated in both miRNA secretomes. It is a negative regulator of the endoplasmic reticulum stress-induced intrinsic apoptotic signaling pathways, allowing cells to survive while stressed. *PRDX4* is downregulated in both miRNA secretomes and is an antioxidant enzyme that neutralizes oxygen species, protects cells against oxidative stress, and is a key protein regulating cellular redox homeostasis. This can be linked to both miRNAs upregulating oxidative stress in breast cancer. Previously, we have shown that treating poorly metastatic tumor cell MCF7 with cell-free conditioned media from miRNA-overexpressed cells enhanced oxidative stress [10].

Next, the eight secretome markers were analyzed to determine significantly enriched GO functions. This identified four cellular components (Fig. 4B); four biological processes (Fig. 4C); one molecular function, phosphoprotein binding (Fig. 4D); and 30 different cell regulatory pathways (Figure S2). The top ten pathways are shown in Fig. 4E. All identified cellular components are related to the extracellular region, confirming these secretome marker roles in TME regulation. Additionally, three biological processes are all related to apoptotic regulation, and one is involved in redox reactions. These processes are altered by miR526b and miR655 in breast cancer. The most significantly enriched pathway identified is FLT3 signaling, which is involved in the cell differentiation, proliferation, and survival of dendritic and hematopoietic progenitor cells [18]. Downstream of FLT3 is the MAPK pathway, which is also a significant pathway listed on the GO function. The MAPK pathway responds to various extracellular stimuli to regulate intracellular processes such as metabolism, proliferation, and apoptosis [18]. The RAF/MAP kinase cascade is highly mutated in cancer, RAS mutations are found in ∼30% of all human cancers, and the most active activator of this pathway is BRAF, which is reported to be mutated in ∼7% of cancers, including breast cancer [19]. FOXO transcription factors (FOXO1, FOXO3, FOXO4) bind to 14-3-3 proteins, such as YWHAB and SFN, allowing their retention in the cytosol [20]. In this study, SFN and YWHAB are upregulated in both miRNA secretomes, indicating the involvement of both markers in cell proliferation and survival. Activation of BAD and translocation to mitochondria are sequestered by 14-3-3 proteins after Akt1 phosphorylation [20]. We reported earlier that expression and function of both miR526b and miR655 are regulated by the PI3K/Akt signaling pathway in breast cancer [8,9].

### 2.5 Validation of secretome markers gene expression in MCF7-miRNA-high cells

**Fig. 5:**
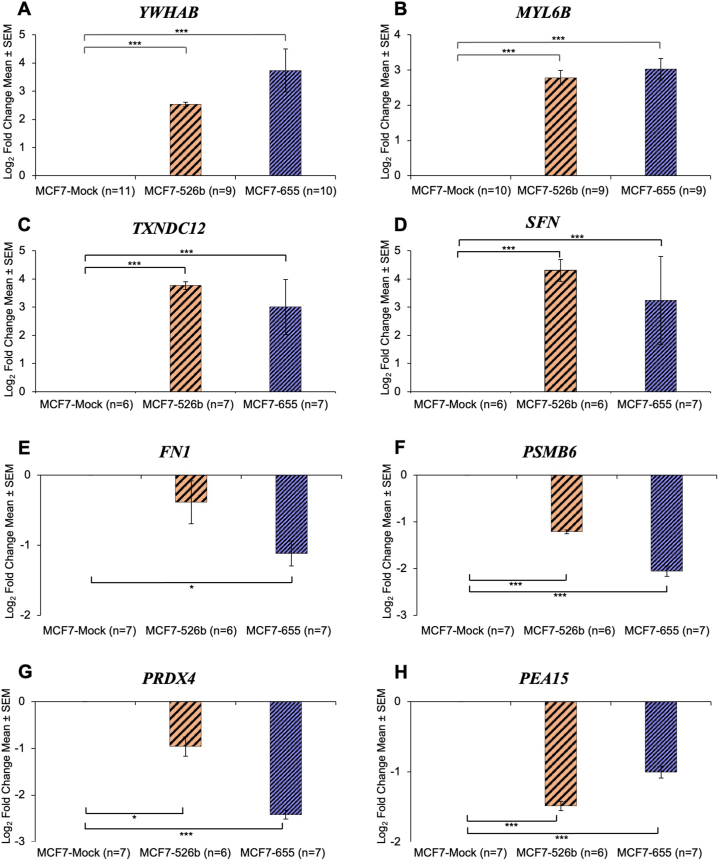
Validation of secretome marker gene expression in MCF7-miR526b and MCF7-miR655 cells compared to MCF7-Mock in total cellular mRNA level. Upregulated secretory marker gene expression was high in both miRNA-overexpressed cells (**A**) *YWHAB,* (**B**) *MYL6B*, (**C**) *TXNDC12*, and (**D**) *SFN.* Downregulated secretory markers gene expression was low in miRNA-overexpressed cells (**E**) *FN1,* (**F**) *PSMB6*, (**G**) *PRDX4*, and (**H**) *PEA15.* The control gene used was *ACTB*.

Secretome marker gene expression in MCF7-miR526b and MCF7-miR655 was analyzed to determine if changes in gene expression are similar to secretory protein expression. All upregulated secretome markers are significantly upregulated in MCF7-miR526b and MCF7-miR655 cells at the mRNA level compared to the MCF7-Mock. There is a log_2_ FC of 2.53 and 3.73 in *YWHAB* expression (Fig. 5A), a log_2_ FC of 2.78 and 3.02 in *MYL6B* expression (Fig. 5B), a log_2_ FC of 3.77 and 3.01 in *TXNDC12* expression (Fig. 5C), and a log_2_ FC of 4.31 and 3.23 in *SFN* expression (Fig. 5D), for MCF7-miR526b and MCF7-miR655 respectively.

All markers downregulated in miRNA-high cell secretions at the protein level are also downregulated in MCF7-miRNA-high cells at the mRNA level. All markers are significantly downregulated in miRNA-overexpressed cells, except for *FN1* in MCF7-miR526b, with a log_2_ FC of −0.38. However, in MCF7-miR655 cells, *FN1* expression is significantly lower, with a log_2_ FC of −1.12 (Fig. 5E). In addition, there is a log_2_ FC of −1.21 and −2.06 in *PSMB6* expression (Fig. 5F)*,* a log_2_ FC of −0.96 and −2.42 in *PRDX4* expression (Fig. 5G), and a log_2_ FC of −1.49 and −1.00 in *PEA15* expression (Fig. 5H), for MCF7-miR526b and MCF7-miR655 respectively. Overall, all markers showed the same pattern of mRNA expression as protein secretions in MCF7-miR526b and MCF7-miR655 cells.

### 2.6 Secretome markers gene expression in cell-free secretions

**Fig. 6:**
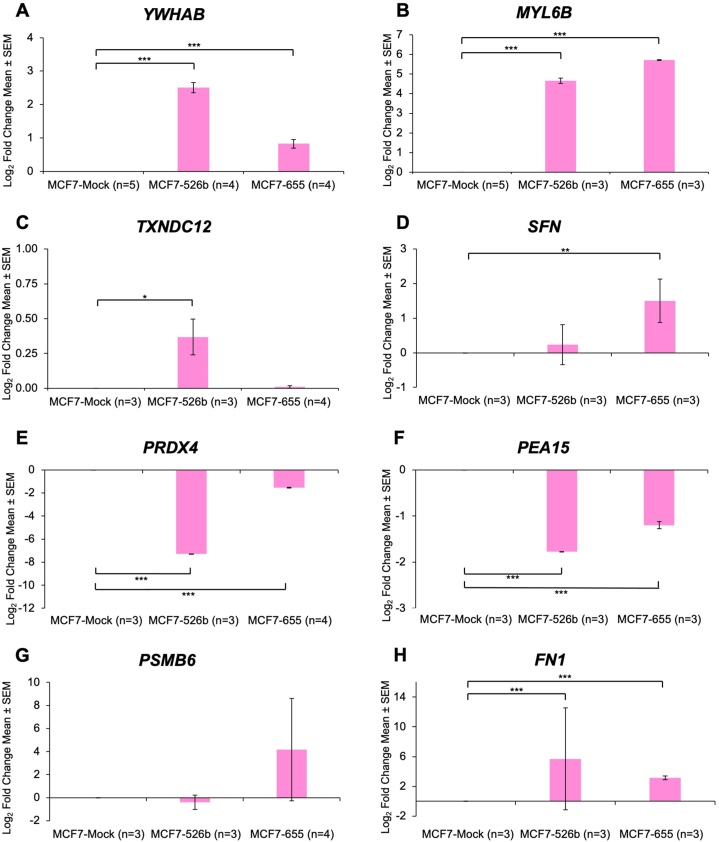
Validation of secretome marker mRNA expression in cell-free secretions. All four upregulated secretory markers mRNA expression in the cell-free secretion (**A**) *YWHAB,* (**B**) *MYL6B*, (**C**) *TXNDC12*, and (**D**) *SFN* was high in miRNA-overexpressed cells. Among four identified downregulated secretory markers, (**E**) *PRDX4* and (**F**) *PEA15* are downregulated in both miRNA cell-free secretions. (**G**) *PSMB6* is downregulated in MCF7-miR526b cell-free secretions but not in MCF7-miR655. (**H**) *FN1* is upregulated in both miRNA cell-free secretions. The control gene used was *RPL5*.

Next, RNA was extracted from the cell-free secretions of all cell lines, and secretome markers were measured at the mRNA level in MCF7-Mock compared to miR526b- and miR655-overexpressing MCF7 cell lines. All upregulated secretome markers are significantly upregulated in cell-free secretions except *TXNDC12* in MCF7-miR655 and *SFN* in MCF7-miR526b cells. There is a log_2_ FC of 2.51 and 0.83 in *YWHAB* expression (Fig. 6A), a log_2_ FC of 4.66 and 5.72 in *MYL6B* expression (Fig. 6B), a log_2_ FC of 0.37 and 0.01 in *TXNDC12* expression (Fig. 6C), and a log_2_ FC of 0.23 and 1.50 in *SFN* expression (Fig. 6D), for cell-free secretions of MCF7-miR526b and MCF7-miR655 respectively.

Among the downregulated secretory protein markers, *PRDX4* and *PEA15* are significantly downregulated in MCF7-miRNA-high cell-free secretions. In MCF7-miR526b, there is a log_2_ FC of −7.29 log_2_ in *PRDX4* expression, and a log_2_ FC of −1.77 in *PEA15* expression (Fig. 6*E*–F). In MCF7-miR655, there is a log_2_ FC of −1.55 in *PRDX4* expression, and a log_2_ FC of −1.20 in *PEA15* expression. *PSMB6* is only marginally downregulated in MCF7-miR526b, with a log_2_ FC of −0.41 (Fig. 6G). *FN1* is upregulated in both MCF7-miR526b and MCF7-miR655 cell-free secretions with a log_2_ FC of 5.69 and 3.15 respectively (Fig. 6H). Overall, all identified upregulated secretory markers and two downregulated markers, *PRDX4* and *PEA15*, showed the same expression pattern at the transcript level, as their respective protein abundance in miRNA-high cell-free secretions.

### 2.7 In silico analysis of miRNA regulating secretome markers gene expressions

**Fig. 7:**
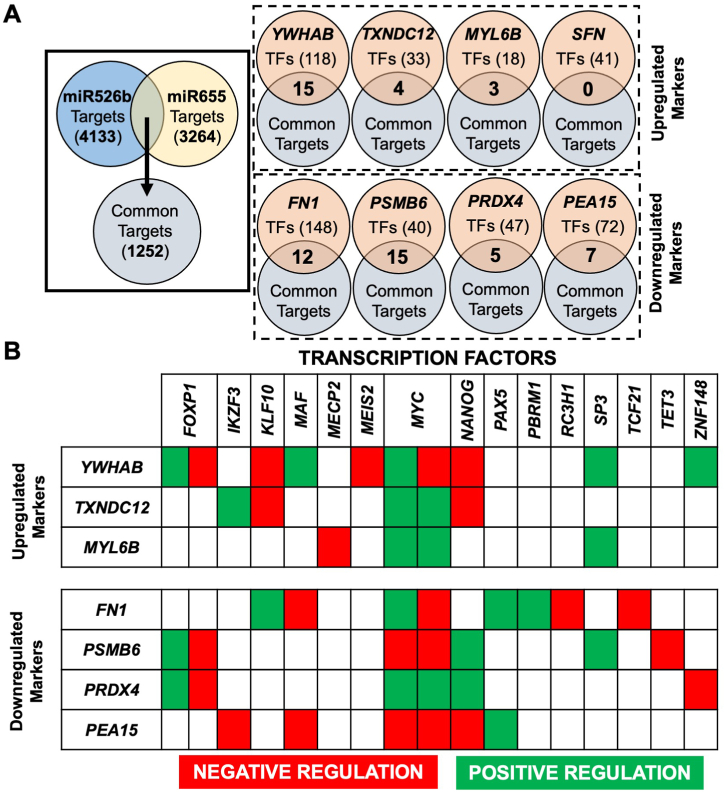
miRNA regulates the transcription factor (TF) of each identified secretory marker. (**A**) Predicted targets of miR526b and miR655 were cross-referenced with predicted TFs of secretome markers. The number in the middle of the Venn diagram shows common TFs which are targets of miRNA. (**B**) Regulatory effects of TFs on secretome markers. Red: Negative regulator TFs, downregulation of these TFs will increase gene expression. Green: Positive regulator TFs, downregulation of these TFs will reduce gene expression. *FOXP1* and *MYC* have been shown to have both positive and negative regulatory functions.

To establish a regulatory connection between miRNA and differential secretion of peptides, known transcription factors (TFs) of the eight markers were cross-referenced with predicted targets of miRNAs. Altogether, miR526b has 4133 predicted targets, and miR655 has 3264 predicted targets, of which 1252 are common targets of both miRNAs (Fig. 7A). Of all targets, *TXNDC12* is a predicted direct target of miR655, and a predicted indirect target of miR526b. However, *TXNDC12* is upregulated in both miRNA secretomes and at the transcript level. This indicates a post-transcriptional regulation of this gene. Therefore, transcription factors negatively regulating *TXNDC12* were investigated, identifying common targets of both miRNAs are *NANOG* and *KLF10* (Fig. 7B). It is plausible that miRNA downregulates negative regulator TFs *NANOG* and *KLF10*, and the absence of these TFs resulted in the upregulation of *TXNDC12* expression. To establish a regulatory connection between miRNA and secretory markers, the indirect relationship between miRNA and marker's TFs was evaluated. For example, upregulated secretome marker *MYL6B* has two positive regulation TFs, *MYC* and *SP3*, and one negative regulation TF, *MECP2*, and all these TFs are targets of miR526b and miR655. Since *MYL6B* is upregulated in both miRNAs' secretomes, we predict that *MECP2* is targeted by both miRNAs and is downregulated. The absence of a negative regulator leads to *MYL6B*'s increased gene and protein expressions. Alternatively, downregulated secretome marker *PRDX4* has three positive regulation TFs, *FOXP1*, *MYC*, and *NANOG*, and two negative regulation TFs, *FOXP1* and *ZNF148*, that are predicted targets of both miRNAs. Since *PRDX4* is downregulated in miRNA-high secretomes, hypothetically, all three positive regulation TFs could be targeted by miR526b and miR655, resulting in *PRDX4* downregulation. There are no TFs of *SFN* that are common predicted targets of both miRNAs. Individually however, seven predicted miR526b targets (Figure S3A), and six predicted miR655 targets (Figure S3B) are TFs regulating *SFN*. In MCF7-miR526b, *SFN* could be upregulated through the downregulation of one or more negative regulation TFs, *ETS2*, *SOX4*, *THRB*, *ESR1*, and *POU5F1*. Similarly, in MCF7-miR655, *SFN* could be upregulated via miR655 targeting negative regulator TFs, *THRA*, *SRF*, and *HNF4A*. These predicted targets need to be further validated.

## 3 Translational validation of secretome markers in silico

### 3.1 Immunohistochemistry analysis of secretome markers

**Fig. 8:**
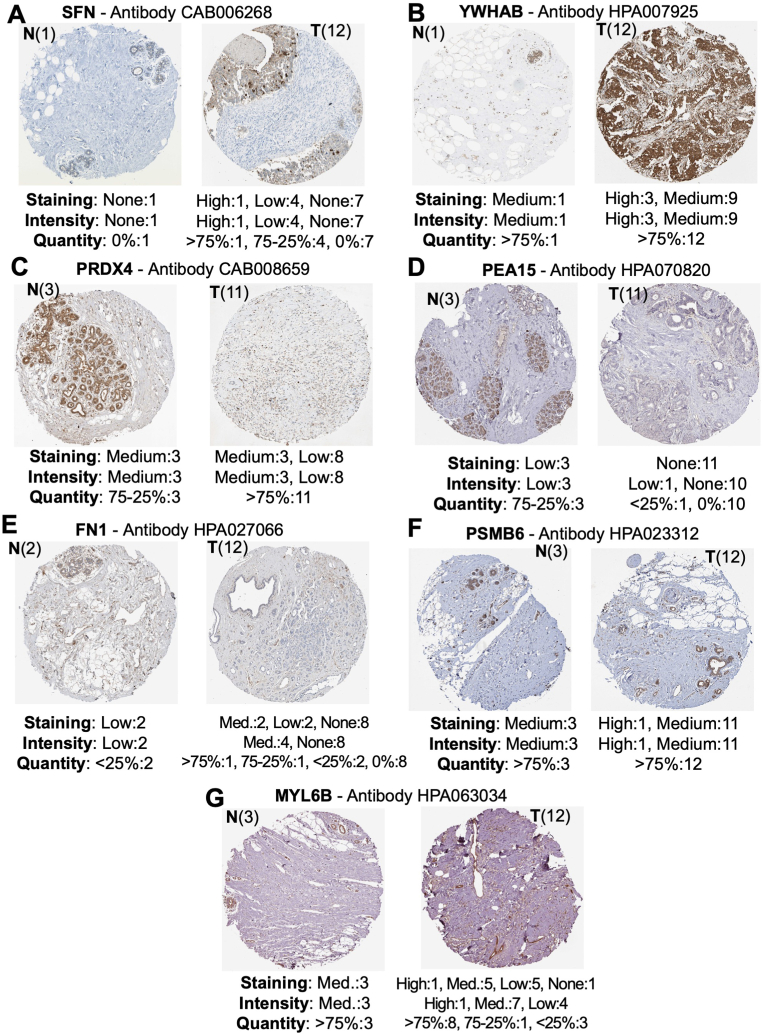
Secretome markers immunohistochemical staining data. Immunohistochemistry staining data for secretome marker (**A**) SFN. (**B**) YWHAB. (**C**) PRDX4. (**D**) PEA15. (**E**) FN1. (**F**) PSMB6. (**G**) MYL6B. TXNDC12 has no immunohistochemistry data available. The brown staining indicates where an antibody has been bound to its corresponding antigen, and blue represents counterstaining.

We wanted to further investigate the presence of these secretory markers within human breast tissue, so an immunohistochemistry analysis was conducted. This also confirms the protein expression of secretome markers in breast tissue compared to normal tissue. One to three normal tissues and 10–12 tumor tissue data were available in the Human Protein Atlas (HPA). For SFN, which is upregulated in the secretome, five of 12 breast tumor tissues show high or low positive intensity staining, compared to normal tissue, which did not show any staining (Fig. 8A). All breast tumor samples stained positive for YWHAB (at high or medium levels) compared to medium staining in normal tissue (Fig. 8B), thus confirming secretome results. For PRDX4, eight of 11 samples show low staining in tumor tissue, compared to medium intensity in all normal samples (Fig. 8C). PEA15 protein is downregulated in both miRNA secretomes, interestingly, in HPA data, no breast cancer tissues showed PEA15 expression, but all three control samples showed low PEA15 expression (Fig. 8D), supporting PEA15 secretome expression. Another downregulated secretome marker, FN1, showed no staining in breast tumor tissue, while normal tissues showed low-intensity staining (Fig. 8E). PSMB6 and MYL6B have the same expression in breast tumors and normal tissues (Fig. 8F–G). TXNDC12 has no immunohistochemistry data available. Overall, SFN, YWHAB, PRDX4, FN1, and PEA15 immunohistochemistry data supported the trends seen in the secretome data.

### 3.2 Gene expression analysis in breast cancer tissue

**Fig. 9:**
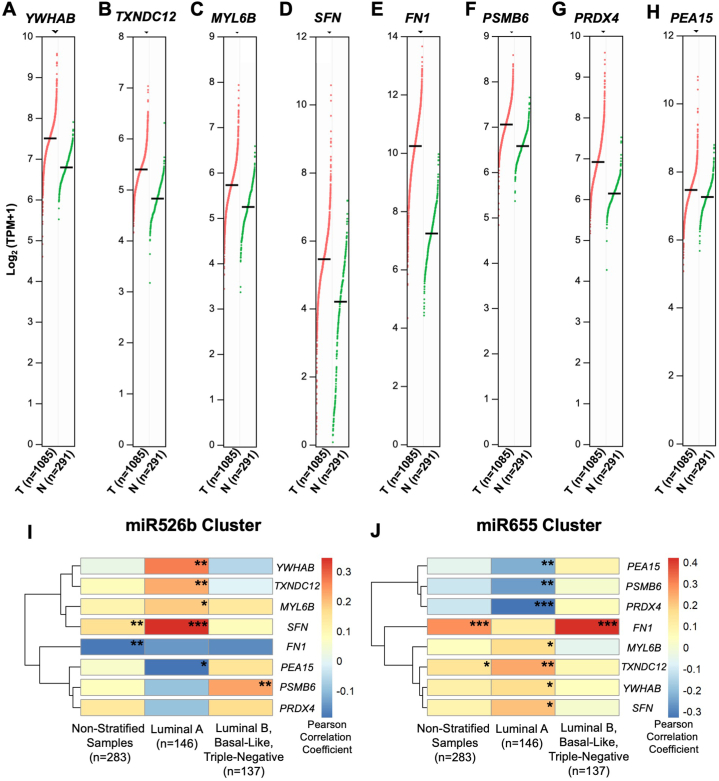
Gene expression in breast tissues and measurement of correlation between gene and miRNAs clusters. Gene expression in human breast tumor and normal tissue for (**A**) *YWHAB*, (**B**) *TXNDC12*, (**C**) *MYL6B*, (**D**) *SFN*, (**E**) *FN1*, (**F**) *PSMB6*, (**G**) *PRDX4*, and (**H**) *PEA15*. (T): Breast cancer tumor samples. (N): Normal breast samples. The black line represents the median expression value, and each dot is a patient sample. Pearson's correlation coefficient heatmap for secretome markers in breast cancer subtypes for (**I**) miR526b cluster and (**J**) miR655 cluster. Red: strong positive correlation. Blue: strong negative correlation. *p < 0.05, **p < 0.01, ***p < 0.001.

Next, the expression of the eight secretome markers were analyzed (data extracted from GEPIA2) in breast tumor tissue (n = 1085) compared to normal tissues (n = 291) to investigate whether their secretory protein expression matches mRNA expression in breast tumor tissue. mRNA expression was measured at the level of Transcripts Per Million (TPM), log_2_ (TPM+1). *YWHAB* is found at 7.51 log_2_ (TPM+1) in breast tumor tissue compared to 6.80 log_2_ (TPM+1) in normal tissue samples (Fig. 9A). In tumor tissue, *TXNDC12* is 5.40 log_2_ (TPM+1) in comparison to 4.83 log_2_ (TPM+1) in normal tissue (Fig. 9B). *MYL6B* is 5.74 log_2_ (TPM+1) in tumor tissue in contrast to 5.25 log_2_ (TPM+1) in normal tissue (Fig. 9C). In tumor tissue, *SFN* expression is measured at 5.47 log_2_ (TPM+1) compared to 4.21 log_2_ (TPM+1) in normal tissue (Fig. 9D). However, downregulated secretome markers *FN1*, *PSMB6*, *PRDX4*, and *PEA15* are also upregulated at the mRNA level in breast tumors (Fig. 9*E*–H). The observed downregulation of secretome markers in miRNA-high secretomes could be due to miRNA targeting positive regulator TFs of markers. All upregulated secretory protein markers show the same expression trends in breast cancer tumor tissue at the mRNA level.

### 3.3 Correlation between secretome markers gene expression and miRNAs cluster expressions in different breast cancer subtypes

To determine the level of correlation between miRNA and secretome markers in different breast cancer subtypes, miR526b and miR655 cluster expressions and secretome markers mRNA expression data were extracted from cBioportal. 283 matched tumor tissue samples were available for both miRNA clusters expression and secretome markers mRNA expression (Fig. 9I–J). The Pearson correlation coefficient between miRNA cluster expression and marker mRNA expression was measured in non-stratified tumor samples, the luminal A subtype, and all remaining tumor subtypes.

In non-stratified samples, miR526b cluster expression showed a positive correlation with *SFN* and a negative correlation with *FN1* (Fig. 9I), confirming secretome results. However, in the luminal A subtype, all four upregulated secretome markers, *YWHAB*, *TXNDC12*, *MYL6B,* and *SFN*, have a significant positive correlation with miR526bs cluster and downregulated secretome marker *PEA15* showed a significant negative correlation, which supports the secretome results.

For miR655 cluster, in non-stratified samples, *TXNDC12* and *FN1* are positively correlated with the miR655 cluster (Fig. 9J). In the luminal A subtype, *YWHAB*, *MYL6B*, *TXNDC12,* and *SFN* showed a significant positive correlation to the miR655 cluster, and *PSMB6, PRDX4,* and *PEA15* showed a significant negative correlation, which supports secretome results. Overall, four upregulated secretome markers (*YWHAB*, *SFN*, *TXNDC12*, *MYL6B*) showed a statistically significant positive correlation and one downregulated secretome marker (*PEA15)* showed a statistically significant negative correlation with both miRNA clusters in luminal A tumors. Hence, these markers play a key role in miRNA-induced tumor metastasis in luminal A breast cancer.

### 3.4 Secretome markers associated with breast cancer patient survival

**Fig. 10:**
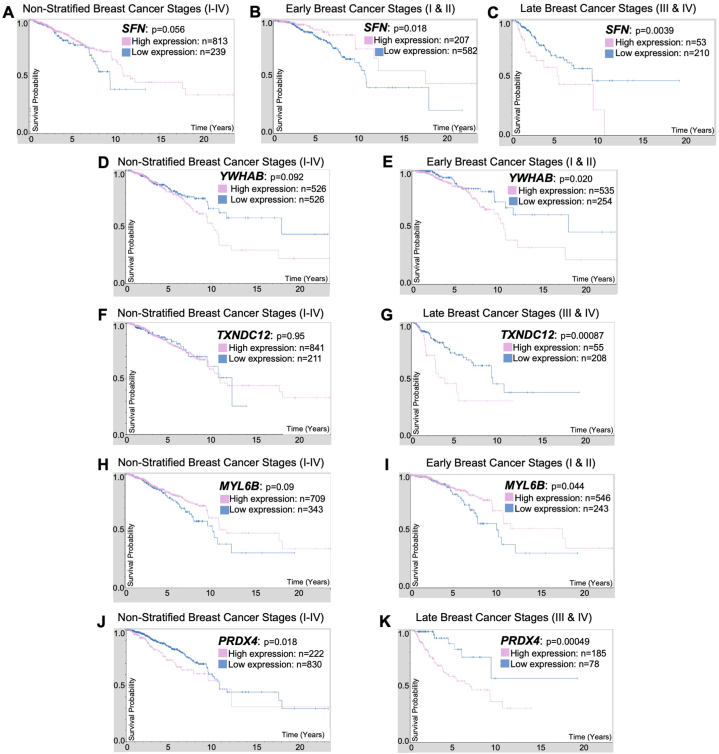
Secretome markers Kaplan-Meier survival plots. *SFN* in (**A**) non-stratified, (**B**) early-stage (stages I-II), and (**C**) late-stage (stages III-IV) breast cancer. *YWHAB* in (**D**) non-stratified and (**E**) early-stage breast cancer. *TXNDC12* in (**F**) non-stratified and (**G**) late-stage breast cancer. *MYL6B* in (**H**) non-stratified and (**I**) early-stage breast cancer. *PRDX4* in (**J**) non-stratified and (**K**) late-stage breast cancer. *PEA1*5 has no data. The x-axis shows time (years), and the y-axis represents survival probability, where 1.0 equals 100%.

Kaplan-Meier survival analysis was conducted using HPA data for secretome markers in non-stratified (all stages I-IV), early (stages I & II), and late stages (III & IV) tumor samples to examine if the expression of any secretome marker is associated with poor patient survival (Fig. 10). Decreased *SFN* expression led to poor survival in non-stratified samples (*p* = 0.056) (Fig. 10A) and significantly reduced survival in early stages (*p* = 0.018) (Fig. 10B). In contrast, in late tumor stages, high *SFN* expression led to significantly reduced survival (*p* = 0.0039) (Fig. 10C), indicating the association of *SFN* with tumor progression. High *YWHAB* expression showed marginally reduced survival in non-stratified samples (*p* = 0.092) (Fig. 10D) but significantly reduced survival in early-stage tumors (*p* = 0.020) (Fig. 10E). Hence, it is a marker associated with the early onset of disease. *TXNDC12* expression is not correlated with breast cancer patient survival in non-stratified samples (*p* = 0.95) (Fig. 10F). However, high *TXNDC12* expression in late-stage tumors is significantly associated with poor survival (*p* = 0.00087) (Fig. 10G), confirming its role in tumor metastasis. Low *MYL6B* expression showed slightly reduced survival in non-stratified samples (*p* = 0.09) (Fig. 10H) and significantly reduced survival in early stages (*p* = 0.044) (Fig. 10I), considering this is a marker associated with cell adhesion, low expression of *MYL6B* in early stages might promote metastasis. High *PRDX4* expression led to significantly decreased survival in non-stratified samples (*p* = 0.018) (Fig. 10J), and also in late stages (*p* = 0.00049) (Fig. 10K), this marker is associated with oxidative stress, conforming its associated with breast cancer progression and metastasis. Overall, *SFN*, *YWHAB*, *TXNDC12*, *MYL6B*, and *PRDX4* expressions are significantly associated with breast cancer patient survival. Detailed survival analysis for all markers in all stages can be found in Figure S4.

### 3.5 Secretome marker expression in blood

**Fig. 11:**
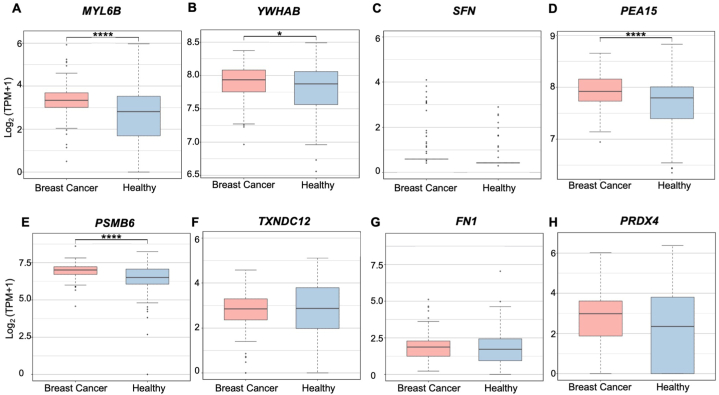
Secretome marker mRNA expression in breast cancer patient blood (n = 140) and healthy participant blood (n = 118) for (**A**) *MYL6B*, (**B**) *YWHAB*, (**C**) *SFN*, (**D**) *PEA15*, (**E**) *PSMB6*, (**F**) *TXNDC12*, (**G**) *FN1*, and (**H**) *PRDX4*. *p < 0.05, ****p < 0.0001.

The mRNA expression of each secretome marker was explored in the blood exosomes of breast cancer patients (n = 140) compared to healthy controls (n = 118) to examine their potential as blood-based biomarkers (Fig. 11). Data was extracted from ExoRBase2.0.

*MYL6B* expression is significantly upregulated in breast cancer patient blood at 3.42 log_2_ (TPM+1) compared to 3.06 log_2_ (TPM+1) in healthy participants’ blood samples (Fig. 11A). In breast cancer patient blood, *YWHAB* has significantly higher expression at 7.91 log_2_ (TPM+1) compared to 7.84 log_2_ (TPM+1) in healthy samples (Fig. 11B). *SFN* is determined to be 0.61 log_2_ (TPM+1) in cancer samples blood in contrast to 0.47 log_2_ (TPM+1) in healthy samples (Fig. 11C). *PEA15* and *PSMB6* are significantly upregulated in cancer samples compared to healthy controls, but this is opposite to their secretory protein expression (Fig. 11D–E). *TXNDC12*, *FN1*, and *PRDX4* have conflicting expressions in breast cancer patient blood compared to control samples in relation to secretory protein expression (Fig. 11F–H). Thus, only *MYL6B*, *YWHAB*, and *SFN* expression in breast cancer blood reflected secretory protein expression.

Additionally, using the Human Proteome Organizations (HUPO) and Human Proteome and Plasma Proteome Projects data, all eight secretome markers could be found in human blood at the highest level of protein evidence (protein level) and the highest level of certainty (canonical) (Table S2). This indicates all these markers are potential blood biomarkers. A summary of secretome markers validated in vitro and passed bioinformatics assays is shown in Table 1. These results show evidence of identified markers’ roles as breast cancer tumor markers associated with TME regulation, and selective secretome markers should be further investigated in various tumor stages and grades in blood samples to confirm their potential as biomarkers.

**Table 1:**
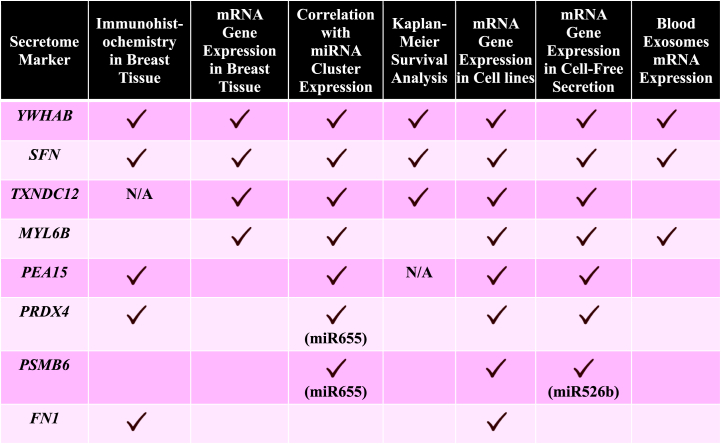
Summary of secretome markers in vitro and bioinformatic translational assays in breast cancer that abided by protein expression from this study. *YWHAB* and *SFN* passed all in vitro and bioinformatic analysis.

## 4 Discussion

We have identified that cell-free miRNAs miR526b and miR655, and miRNA-overexpressed tumor cell secretory proteins change cellular phenotypes in the TME. Therefore, analysis of miR526b- and miR655-overexpressing cell secretomes in the luminal A MCF7 breast cancer cell line might help decipher the mechanisms of miRNA regulating the TME and identify breast cancer biomarker candidates. However, extensive secretome analysis can impose difficulties, as many extracellular proteins are signaling molecules found at low levels, and thus, some molecules of interest may be lost during data curation and analysis. Our platform combining nano-high-performance liquid chromatography with large-sensitivity mass spectrometry ensured an in-depth, sensitive secretome analysis. After systematic data curation, four upregulated (*YWHAB*, *TXNDC12*, *MYL6B*, *SFN*) and four downregulated (*FN1*, *PSMB6*, *PRDX4*, *PEA15*) markers in miRNA-overexpressed tumor secretomes were identified.

miR526b and miR655 have been shown to induce oxidative stress by overproduction of ROS and promoting ROS levels during hypoxia in miRNA-overexpressed cells [10,12]. Overproduction of ROS disrupts tissue homeostasis, causes DNA damage, and often triggers apoptosis. The hypoxic core in a growing tumor influences the apoptotic pathways; however, in miR526b and miR655-overexpressed breast cancer cells, hypoxia further promotes oxidative stress, cell migration, and tube formation [12]. Here, we show that the most enriched biological processes regulated by the eight secretome markers are related to apoptosis regulation and cell redox homeostasis. Secretome markers may regulate ROS levels, cellular response to hypoxia, and apoptosis in favor of tumor cell survival, supporting miR526b and miR655 as mediators of these processes in breast cancer.

In our study, YWHAB and SFN are upregulated in both miRNA secretomes. YWHAB and SFN are both members of the 14-3-3 protein family. These proteins regulate cell cycle machinery and signaling pathways and modulate the activity of their binding partners [21]. *YWHAB* has an oncogenic role in cancer [22], and *SFN* (*14-3-3σ*) is typically documented as a tumor suppressor [22], although the roles of *SFN* in breast cancer are not so clear [[23], [24], [25]]. YWHAB is a non-classically secreted protein, and SFN is classically secreted. These 14-3-3 proteins could be secreted in MCF7-miRNA-high cells by altering cell machinery and signaling pathways, as 14-3-3 proteins have an active role in protein transport and can activate the Wnt signaling pathway [26,27]. This, in turn, promotes cell survival during oxidative stress and hypoxia by inhibiting pro-apoptotic pathways stabilizing hypoxia-inducible factor 1 alpha (HIF-1α), allowing the expression of genes that promote EMT and metastasis [20]. Thus, 14-3-3 proteins may promote miR526b- and miR655-induced cell proliferation and EMT mechanisms in the TME.

*YWHAB* and *SFN* are upregulated in MCF7-miRNA-high cells and cell-free secretions at the mRNA level. These two markers show higher mRNA and protein expression in breast tissue compared to non-cancerous tissue. The gene expression of both has positive correlations to miRNA cluster expression in luminal A breast cancer. High *YWHAB* expression could significantly predict a worse prognosis in early-stage breast cancer, and its overexpression in luminal A breast cancer has led to a worse prognosis [28]. So, *YWHAB* is a marker associated with breast cancer metastasis. *SFN* has a tumor suppressor role, so the loss of *SFN* is required to initiate metastasis, and at a progressive stage, mutated *SFN* overexpression might behave more like an oncogene; hence higher expression of SFN promotes metastasis. We found low expression of SFN is associated with early-stage patient overall survival, whereas higher expression is associated with late-stage tumors. Both *YWHAB* and *SFN* are upregulated in breast cancer blood samples and could be found in blood plasma at the highest level of protein certainty. Hence, *YWHAB* and *SFN* are strong blood-based breast cancer biomarker candidates and tools for understanding TME regulation.

TXNDC12 is a classically secreted protein that is upregulated in MCF7-miRNA-high secretomes. Transcripts of *TXNDC12* are significantly upregulated in MCF7-miRNA-high cells and cell-free secretions. This may be attributed to miR526b and miR655 targeting negative regulator TFs *NANOG* and *KLF10* of *TXNDC12*. *TXNDC1*2 has roles in redox regulations, defense against oxidative stress, and regulation of transcription factors TFs [29]. Upregulation of *TXNDC12* inhibits apoptosis by endoplasmic reticulum stress-inducing agents and promotes EMT and metastasis in many epithelial cancers [30,31]. Additionally, since both miRNAs and *TXNDC12* promote oxidative stress and EMT, *TXNDC12* likely collaborates with miRNAs to promote breast cancer cell migration, invasion, and metastasis. While *TXNDC1*2 has not been studied in breast cancer, two gene family members of *TXNDC12*, *ARG2* and *ARG3*, are known serum-based breast cancer biomarkers [32]. In breast cancer tissue, *TXNDC1*2 mRNA expression is higher compared to normal samples and is significantly correlated with both miRNA clusters in luminal A breast cancer. High *TXNDC12* expression could differentiate late-stage metastatic tumors. Thus, *TXNDC12* shows potential as a prognostic biomarker for metastatic breast cancer and a major regulator of miRNA functions.

MYL6B is a classically secreted protein found upregulated in MCF7-miRNA-high cell secretomes. In miRNA-high cells and cell-free secretions, *MYL6B* is significantly upregulated, potentially from both miRNAs targeting negative regulator TF *MECP2*. *MYL6B* is an essential light chain subunit for myosin motor proteins and regulates cell mobility functions [33]. Both miRNA-overexpression enhanced cell migration and invasion of tumor cells and cell migration and angiogenesis properties of HUVEC cells in the TME, so we speculate that MYL6B might be driving these phenotypes for miR526b and miR655. Although no studies have investigated *MYL6B* in breast cancer, *MYL6B* overexpression promotes EMT in rectal adenocarcinoma, and in other epithelial cancers, high *MYL6B* expression is reported as a predictor of poor survival [34]. *MYL6B* mRNA expression is higher in breast tumor tissue than in normal tissue, and in luminal A breast cancer, *MYL6B* expression shows a significant positive correlation with miRNA cluster expression. Similarly, we reported both miRNA expression promotes metastasis in luminal-A breast cancer subtype. *MYL6B* is significantly upregulated in breast cancer patient blood and can be identified at the highest level of protein certainty in human plasma. Thus, the potential of *MYL6B* as a blood-based breast cancer biomarker should be further explored.

*MYC* is a regulator of seven secretome markers except for *SFN*. *MYC* regulates ∼15% of human genes, and in breast cancer, *MYC* targets genes that participate in cancer stem cell (CSC) regulation, angiogenesis, cell growth, and transformation [35]. Both miR526b and miR655 enhanced CSC phenotypes in both luminal A and HER2 positive breast cancer [8,9], and *MYC* expression in MCF7-miR655 cells is marginally upregulated [9]; however, *MYC* is a predicted target of miR655. We speculate that other downstream effector molecules may compensate for *MYC* expression in miRNA-overexpressed cells. *MYC* has been deemed both a positive and negative regulator of *YWHAB* and *FN1,* hence depending on tissue and tumor type, the regulation of MYC varies*.* Thus, the function of *MYC* in the breast cancer secretome in miRNA-high breast tumor cells requires further investigation.

All downregulated secretory markers have decreased cellular gene expression in all miRNA-high cells. One of them, *PEA15*, is downregulated at both nuclear and cell-free secretion mRNA expression levels. This could be due to miRNAs indirectly targeting TFs of respective genes. Three downregulated markers, PEA15, PRDX4, and FN1, also showed low protein expression in breast tumors, as confirmed with immunohistochemistry staining. Low expression of *PEA15* is strongly correlated to both miRNA clusters expression in luminal A breast cancer. Therefore, *PEA15* is a negative regulator of breast cancer; hence it is downregulated in the secretions of MCF7-miR526b and MCF7-miR655.

*PRDX4* protects cells during oxidative stress [36], and hence decreased expression of PRDX4 in miRNA-high tumors secretome could contribute to increased oxidative stress in the TME. Furthermore, *FN1* fragments are known to inhibit angiogenesis [37]. Thus reduced *FN1* expression in miRNA-high cells may add to the angiogenesis-promoting phenotype of miRNA-high cells. The downregulation of four markers in miRNA-secretomes could be related to miRNA epigenetically regulating these marker expressions in breast cancer. Further investigation of downregulated marker expression in breast cancer with miRNA context is needed. All identified secretome markers are prognostic markers in other epithelial cancers and can be detected in the blood. Therefore, identified upregulated secretory markers *YWHAB*, *SFN*, *TXNDC12*, and *MYL6B* show strong potential in establishing a battery of breast cancer biomarkers alongside pri-miR526b and pri-miR655. To our knowledge, this is the first time secretory proteins from miR526b- and miR655-overexpressing ER-positive luminal A cells have been identified and investigated to decipher the mechanisms of miRNA TME regulation. In addition, most secretome marker expressions correlate with miRNA cluster expressions in luminal A breast cancer, strengthening our hypothesis that upregulated secretome markers might be potential blood-based biomarkers for ER-positive luminal A breast cancer.

Many low abundance but vital secretory proteins were excluded during data curation to find common secretory proteins in both miRNA-secretomes. In the future, each MCF7-miRNA-secretome will be investigated separately to identify specific secretory proteins regulated by each miRNA. Additionally, we will further investigate the roles of the eight identified secretome markers in breast cancer progression and metastasis to establish their roles as potential drug targets. Each of the novel secretory markers will be tested in matched breast cancer patient plasma and tissue samples to determine their potential as blood-based breast cancer biomarkers. These secretome markers might increase the sensitivity and specificity of breast cancer early detection in combination with pri-miRNAs, allowing us to develop a battery of blood-based breast cancer biomarkers for luminal A or ER-positive breast cancers.

## 5 Materials and methods

This project is approved by Brandon University Research Ethics (#21986, approved on April 21, 2017) and Biohazard Committee (#2017-BIO-02, approved on September 13, 2017).

### 5.1 Cell culture

Human breast cancer cell line MCF7 was purchased from American Type Culture Collection (ATCC, Rockville, MD, USA). Stable miRNA-overexpressing MCF7-miR526b and MCF7-miR655 and empty vector-transfected cell line MCF7-Mock were established by overexpression of miRNA plasmids as previously described [8,9]. MCF7-Mock, MCF7-miR526b, and MCF7-miR655 were grown in complete RPMI 1640 media (Gibco, ON, Canada) with 10% fetal bovine serum (VWR, ON, Canada) and 1% Pen-Strep. Stable miRNA-overexpressed cells and Mock (empty vector-transfected) cells received Geneticin (G418) at a concentration of 200 ng/ml (Biobasic, ON, Canada) and incubated at 37 °C in humid conditions and with 5% CO_2_. Once cell confluency reached 90%, the cells were harvested for RNA extraction.

### 5.2 Collection of conditioned media for secretome analysis

Once cell confluency reached 90%, all cells were washed with 1*x* phosphate-buffered saline (PBS) (Gibco, ON, Canada) to remove any trace of complete media. Cells were then serum-starved with basal media for 24 h, and the conditioned media, which contains all the secretory proteins and cell metabolites, was collected.

### 5.3 Preparation of proteins to be analyzed by nanoHPLC-MS

Proteins within the conditioned media were precipitated overnight with 35% ethanol, followed by acidification with sodium acetate and the addition of a digestion buffer (1% sodium deoxycholate and 50 mM NH_4_HCO_3_). Precipitated proteins were quantified by BCA Protein Assay Kit (Pierce, Rockford, IL) with at least 100 μg protein per cell line. Peptides were isolated by stage tip purification before analysis by nanoHPLC-MS (Agilent 6530 Accurate-Mass Q-TOF LC/MS, Santa Clara, CA). In a single run, there were two experimental replicates of each sample. So, with three biological replicates, n = 6 sets of data for each cell line was generated.

### 5.4 Identification of secreted protein IDs

NanoHPLC-MS used Mascot Server (version 2.6) to identify peptides [38]. A full scan of peptides was quantified by MS1 filtering, extracted ion chromatogram, and verified by spectral matching (Uniprot human protein reference data file) and amino acid database search (<1% false discovery rate (FDR)). Mass spectrometry raw data files and peptide masses were analyzed using Skyline (version 20.1.0.155), which allowed us to acquire a list of protein IDs [39]. MCF7-miR526b and MCF7-miR655 cells (case) were normalized to MCF7-Mock (control), and Skyline gave their protein IDs with corresponding FCs and p-values.

### 5.5 GO analysis of all differentially expressed miRNA-high proteins

The IDs of all differentially expressed proteins were entered into The Gene Ontology Resource for *Homo sapiens* (release 2021-09-01), and data was extracted [40,41].

### 5.6 Threshold determination and data curation

Volcano plots for differentially expressed secreted protein from Skyline were extracted (version 20.1.0.155) [39]. We established a >1.5/<-1.5 log_2_ FC and >0.3 -log_10_ p-value as the threshold following similar studies with secretome analysis in other diseases [42,43]. There were no statistically significant protein IDs (*p* < 0.05), so the top 92nd percentile of data was considered, which roughly translates to 0.3 -log_10_ p-value. Next, we submitted protein IDs to Uniprot and extracted protein names, primary gene names, and synonyms [44]. One Skyline protein ID was unmapped (no peptide ID found), and 13 Skyline protein IDs had no gene names in Uniprot. Therefore, these Skyline protein IDs were excluded from this study. Gene names were used for all differentially secreted protein IDs. If a protein ID corresponded to the same gene name, and one or both log2 FC and -log_10_ p-value differed, average or mean of all IDs with the same gene name were considered. The log_2_ FC and -log_10_ p-value of gene names were further analyzed to identify proteins that pass through our threshold in at least one miRNA-secretome.

### 5.7 Generation of heatmaps

Rstudio version April 1, 1103 and R version 4.0.3 were used to make gene heatmaps. Heatmap functions with default arguments in R were used to produce the agglomerative hierarchical clustered heatmap.

### 5.8 Breast-specific proteome

The 14,227 human protein-coding genes within the breast-specific proteome were extracted from the HPA (version 20.1) [45] and compared to genes within our secretome threshold.

### 5.9 Secretome prediction methods

Classical secretome prediction method data was retrieved from the HPA (version 20.1) [45] for HPA, MDSEC, Phobius, SignalP, and SPOCTOPUS and compared genes within each method to our list of genes that were within our set threshold.

For the non-classical secretome prediction method, SecretomeP, FASTA sequences of the identified threshold protein IDs were obtained via Uniprot and submitted in SecretomeP 2.0 (December 2020) for mammalian sequences [17,44]. Genes were considered non-classically secreted following the previously established guideline of a neural network score >0.6 and odds >3 [17,46]. Secretome markers were considered classically secreted if found in classical and non-classical secretion methods [17].

### 5.10 Secretome marker functions and GO analysis

Secretome marker general functions were obtained by using www.GeneCards.org version 5.6.0 Build 515 [47]. Individual GO functions of each secretome marker were obtained through Uniprot (Last modified: February 2, 2021) [44] and QuickGo (GO version 2021-11-08) [48]. Shared GO of the eight secretome markers was found by analyzing all protein IDs as one quarry into the Gene Ontology Resource for *Homo sapiens* (release 2021-09-01) [40,41]. This obtained cellular component and Reactome pathway results. Additionally, secretome markers were analyzed with STRING database (version 11.0 b) [49] to identify biological processes and molecular functions GO.

### 5.11 Cellular RNA extraction

Cells were grown to 90% confluency, and RNA extraction was performed with the Qiagen miRNeasy Mini Kit (Qiagen, ON, Canada) following the manufacturer's protocols.

### 5.12 Cell-free RNA extraction

Once cell confluency reached 90%, all cells were washed with 1*x* phosphate-buffered saline (PBS) (Gibco, ON, Canada) to remove any trace of complete media. Cells were then serum-starved with basal media (FBS and penicillin-streptomycin free) for 24 h, and the conditioned media, which contained cell secretion and metabolites, was collected. The media was centrifuged for 5 min at 25 °C and 4000RPM. The supernatant was removed and placed in a new falcon tube; the remaining pellet was discarded. TRIzol was added at the same volume of sample used, and chloroform was added at half the amount of sample used. The falcon tube was centrifuged at 4000 RPM at 4 °C for 15 min. The aqueous layer was collected and combined with 1.5 times the amount of ethanol. RNA was collected using the miRNeasy Mini Kit (Qiagen, ON, Canada), following the manufacturer's protocols for cell-free RNA extraction.

### 5.13 cDNA synthesis

The RNA extracted from cells, and cell-free secretions were reverse transcribed into complementary DNA (cDNA) using the High-Capacity cDNA Reverse Transcription Kit (Applied Biosystems, MA, USA).

### 5.14 Quantitative real-time PCR

For quantitative real-time PCR (qRT-PCR), TaqMan Gene Expression Assays were used, with TaqMan Universal PCR Master Mix (Applied Biosystems, MA, USA) and specific control and experimental probes. All probes and primers are designed following Build GRCh38 (Genome Reference Consortium Human Build 38) by ThermoFisher Scientific, ON, Canada. Ubiquitous control genes *RPL5* (Hs03044958_g1, Chromosome Location: Chr.1: 92,832,037–92,841,924) and *Beta*-*actin* (Hs01060665_g1, Chromosome Location: Chr.7: 5,527,148–5,530,601) were used, and experimental genes *YWHAB* (Hs00793604_m1, Chromosome Location: Chr.20: 44,885,599–44,908,532), *MYL6B* (Hs05005444_g1, Chromosome Location: Chr.12: 56,152,420–56,157,987), *TXNDC12* (Hs00210841_m1, Chromosome Location: Chr.1: 52,020,131–52,056,171), *SFN* (Hs00968567_s1, Chromosome Location: Chr.1: 26,863,142–26,864,456), *FN1* (Hs01549976_m1, Chromosome Location: Chr.2: 215,360,440–215,436,167), *PSMB6* (Hs00382586_m1, Chromosome Location: Chr.17: 4,796,144–4,798,503), *PRDX4* (Hs01056076_m1, Chromosome Location: Chr. X: 23,664,260–23,686,399), and *PEA15* (Hs00269428_m1, Chromosome Location: Chr.1: 160,205,319–160,215,376), to determine gene expression in total cellular RNA level and cell-free secretions of different breast cancer cell lines. We established that *RPL5* is a suitable control gene for cell-free secretions [16]. *RPL5* showed consistent expression in all cell lines compared to *Beta*-*actin* (data not shown), thus, *RPL5* served as a control gene for cell-free secretion qRT-PCR.

### 5.15 miRNA target genes and TF analysis

Predicted miR526b (hsa-mir-526 b) and miR655 (hsa-mir-655) targets were downloaded for both mature five-prime sequences from TargetScanVert (Release 7.1) [50]. Only *TXNDC12* was found to be a predicted direct target of miR655. Therefore, a combined list was created, which included common targets of both miRNAs. The Enrichr database [51] was used to identify the TFs for each secretome marker. Each marker and its TFs were matched against miR526b and miR655 common targets.

### 5.16 Immunohistochemistry analysis

Normal and breast cancer tissues immunohistochemistry staining data were obtained from the HPA (version 20.1) [45]. For each normal sample, adipocyte, glandular cell, and myoepithelial cell parameters were combined (staining, intensity, and quantity), and the median intensity value was considered. TXNDC12 had no immunohistochemistry data available in the HPA. For most secretome markers, data for several antibodies were available. For each secretome marker, the antibody giving the strongest staining signal was selected for analysis. The antibody that gave positive staining signals for most of the tumor samples, and showed signal intensity, was selected for further analysis.

### 5.17 Breast tissue mRNA expression

mRNA expression of the secretome markers in the breast cancer tumor tissue and controls were obtained from GEPIA2 (2018 version 9) [52], with all parameters set as default.

### 5.18 Pearson correlation coefficient analysis

The miRNAs within the miR526b and miR655 clusters were identified through miRBase (release 22.1) [53]. miR526b's cluster had expression data for two miRNAs, miR516a-1, and miR516a-2. miR655's cluster had expression data for 16 miRNAs: miR134, miR154, miR369, miR376c, miR379, miR381, miR382, miR409, miR410, miR411, miR487b, miR495, miR539, miR655, miR758, and miR889. The mean of all miRNA expressions within each cluster was considered as miR526b and miR655 cluster expression. All 19 breast cancer studies reported were included, and secretome markers and miRNAs within miR526b and miR655 clusters were analyzed using cBioPortal (v 3.7.15) [54]. The “mRNA expression (microarray)” and “microRNA expression” data were used. The Pearson correlation coefficient (R) was measured between each secretome marker and miR526b or miR655 cluster expression in all breast cancer tumor subtypes.

### 5.19 Kaplan-Meier survival analysis

Kaplan-Meier survival data were retrieved from the HPA (version 20.1) [45]. Analysis was done on non-stratified (all stages), early-stage (stages I & II; I, IA, IB, II, IIA, IIB), and late-stage (stages III & IV; III, IIIA, IIIB, IIIC, IV) data sets. The cut-off values used for each secretome marker shown in order of non-stratified, early stages, and late stages are as follows: *YWHAB* (107.89, 95.57, and 109.48), *TXNDC12* (15.97, 17.67, and 22.97), *MYL6B* (7.01, 6.76, and 11.15), *SFN* (17.48, 49.56, and 57.21), *FN1* (192.40, 425, and 309), *PSMB6* (50.47, 54.28, and 47.40) and *PRDX4* (62.87, 37.28, and 31.98). *PEA1*5 had no data.

### 5.20 Breast cancer blood plasma analysis

Secretome marker expression in breast cancer blood exosomes versus healthy controls was retrieved from ExoRBase2.0 (version 2.0) [55].

The HUPO website (www.hupo.org) was used to retrieve the “Human Plasma Proteome Project.” PeptideAtlas (build: Plasma Non-Glyco 2017–04 - Mapping 2017-05-12) [56] was also used to confirm secretome markers’ presence in human plasma.

### 5.21 Statistical analysis

False discovery rate (FDR) corrected p-values were calculated by Skyline using the MSstats R package (version 3.13.6) [39]. GO statistics from The Gene Ontology Resource and STRING database used FDR corrected p-values [40,41,49]. Gene expression results were compared using unpaired, two-tailed *t*-tests in GraphPad Prism (Version 9.2.0). Gene expression in normal and breast cancer tissue was measured using the absolute value of fold change cutoff of log_2_ 1 and q-value cutoff of 0.01 (ANOVA) [52]. miRNA cluster and secretome marker correlation were measured using the Pearson Correlation coefficient. Significant differences between Kaplan-Meier survival curves were determined with the log-rank test [45]. Gene expression in blood exosomes between two groups was compared using an unpaired, two-tailed *t*-test [55].

### 5.22 Data availability

The mass spectrometry proteomics data have been deposited to the ProteomeXchange Consortium via the PRIDE partner repository with the dataset identifier PXD031771 [57]. Breast-specific proteome data was extracted from the HPA (version 20.1) [45]. Classical secretome prediction method data was extracted from the HPA (version 20.1). Non-classical secretome prediction method data was completed in SecretomeP 2.0 (December 2020) [17]. miR526b and miR655 targets were identified through TargetScanVert (Release 7.1) [50]. Enrichr identified the TFs of secretome markers [51]. miR526b and miR655 clusters were identified through miRBase (release 22.1) [53]. Breast cancer and normal immunohistochemistry staining data were obtained from the HPA (version 20.1) [45]. mRNA expression of secretome markers in breast tumors and controls was collected from GEPIA2 (2018 version) [52]. miRNA cluster expression and secretome marker mRNA expression were obtained through cBioPortal (v 3.7.15) [54]. Kaplan-Meier survival data were found from the HPA (version 20.1) [45]. Secretome marker expression in breast cancer blood exosomes and healthy controls were collected from ExoRBase2.0 (version 2.0) [55]. Computer code and all other data supporting the findings of this study are available from the corresponding author upon request.

## Author contribution statement

Riley Feser: Performed the experiments; Analyzed and interpreted the data; Wrote the paper.

Reid M Opperman; Braydon Nault: Analyzed and interpreted the data.

Sujit Maiti: Analyzed and interpreted the data; Contributed reagents, materials, analysis tools or data.

Vincent C Chen: Contributed reagents, materials, analysis tools or data.

Mousumi Majumder: Conceived and designed the experiments; Wrote the paper.

## Data availability statement

Data associated with this study has been deposited at The mass spectrometry proteomics data have been deposited to the ProteomeXchange Consortium via the PRIDE partner repository with the dataset identifier PXD031771 (Username: reviewer_pxd031771@ebi.ac.uk and Password: Pa5Hr3Fk).

## Declaration of competing interest

The authors declare that they have no known competing financial interests or personal relationships that could have appeared to influence the work reported in this paper.

## Appendix A Supplementary data

Multimedia component 3 Multimedia component 3

figs1 figs1

figs2 figs2

figs3 figs3

figs4 figs4

figs5 figs5

figs6 figs6
