# Supplementary figures and images for: Breast cancer cell secretome analysis to decipher miRNA regulating the tumor microenvironment and discover potential biomarkers
Source: Heliyon. 2023 Apr 14;9(4):e15421. doi: 10.1016/j.heliyon.2023.e15421 (PMC10148110; doi:10.1016/j.heliyon.2023.e15421)

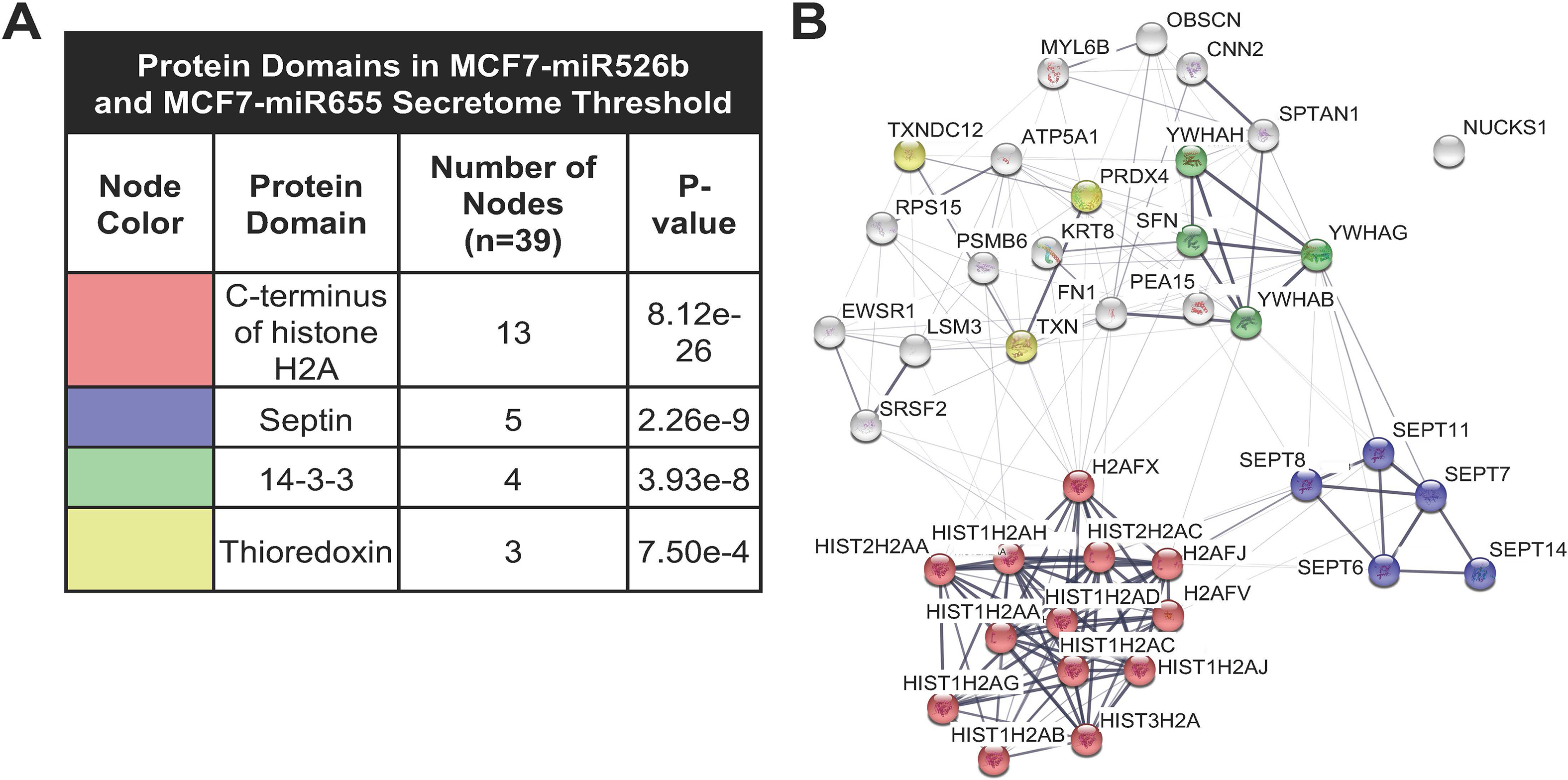

Supplement: figs1 [file mmcfigs1.jpg]

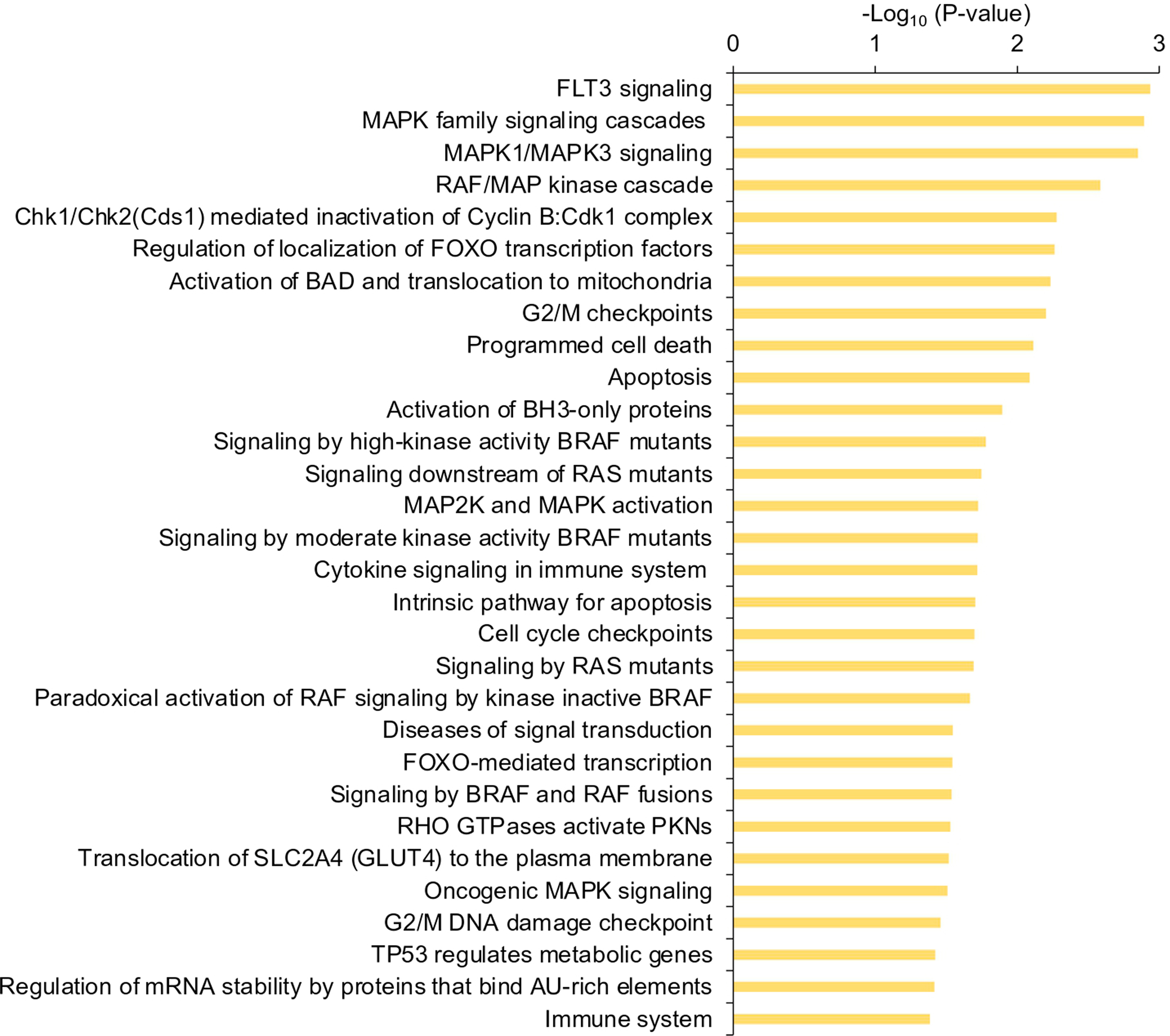

Supplement: figs2 [file mmcfigs2.jpg]

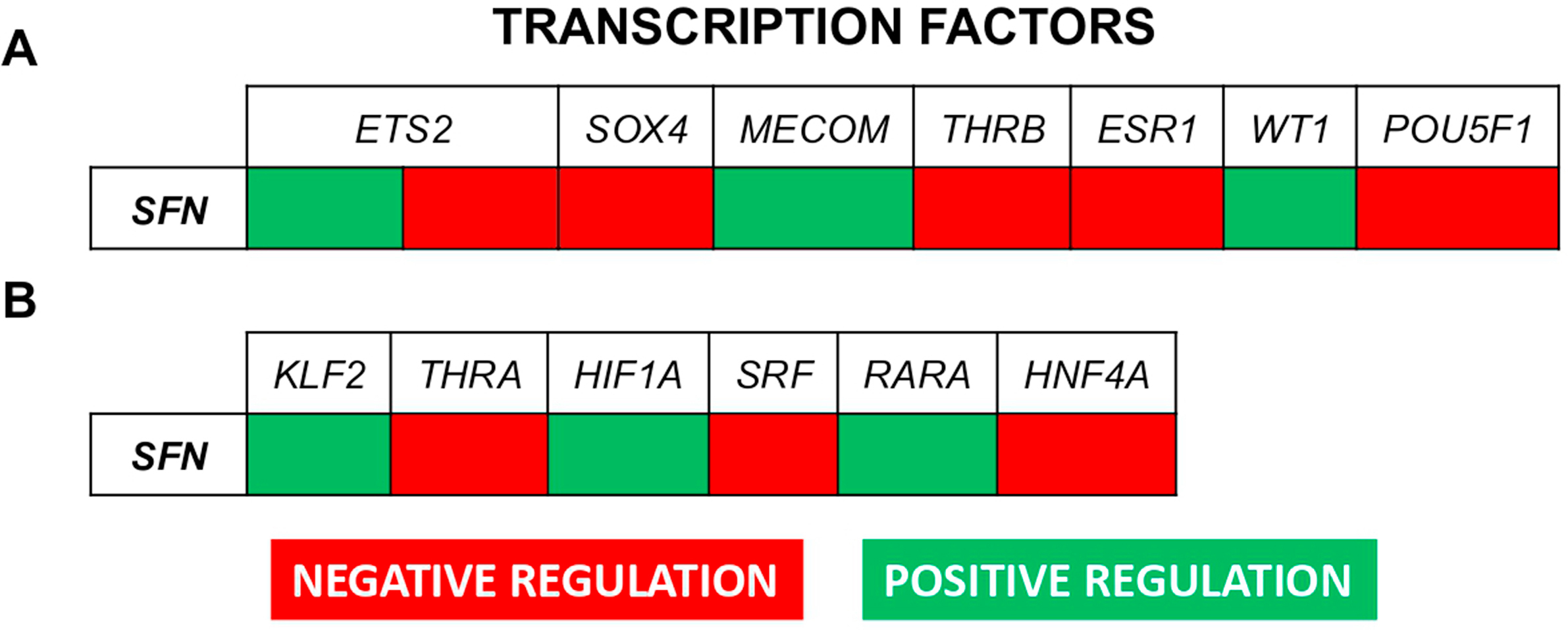

Supplement: figs3 [file mmcfigs3.jpg]

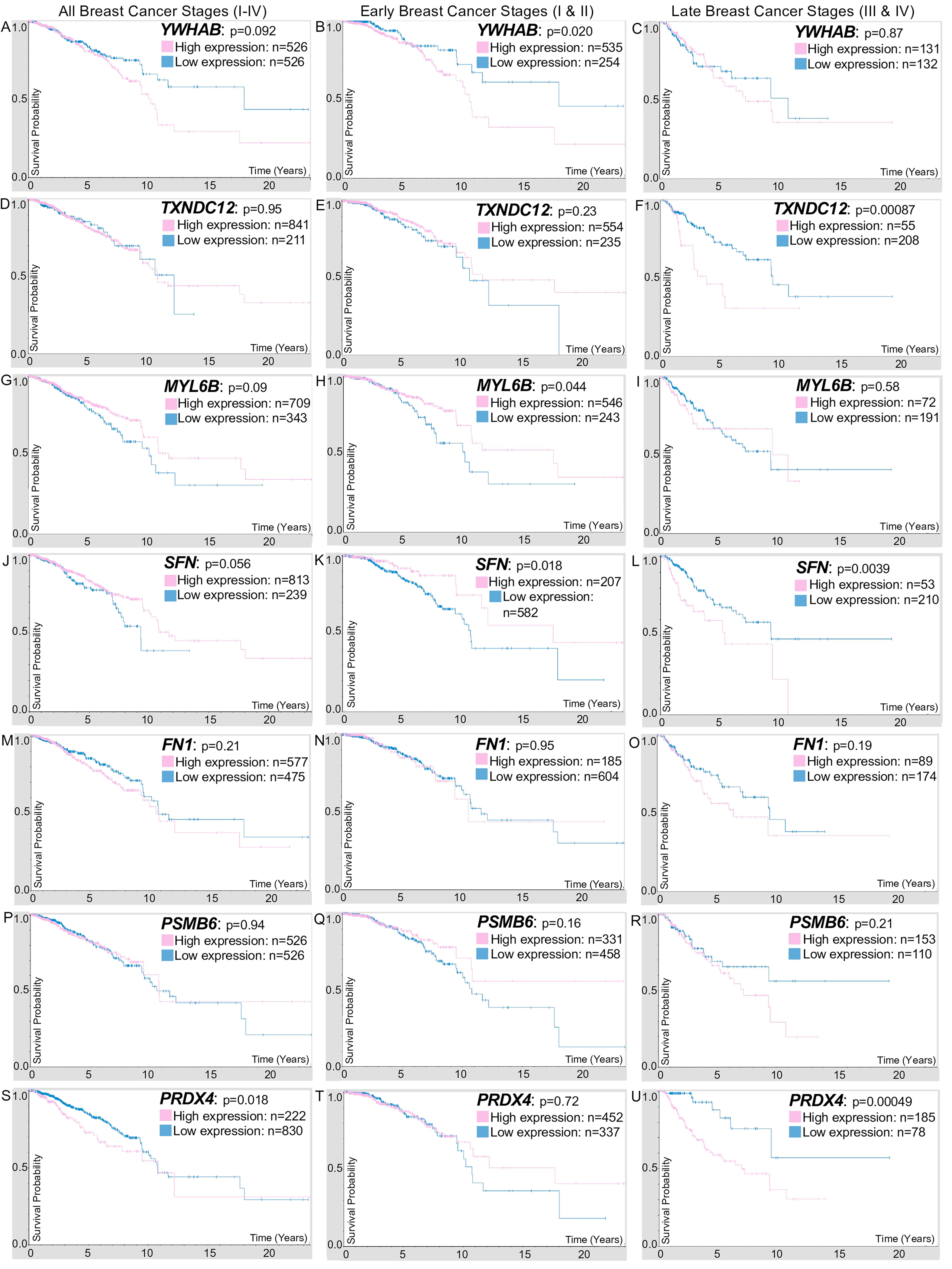

Supplement: figs4 [file mmcfigs4.jpg]

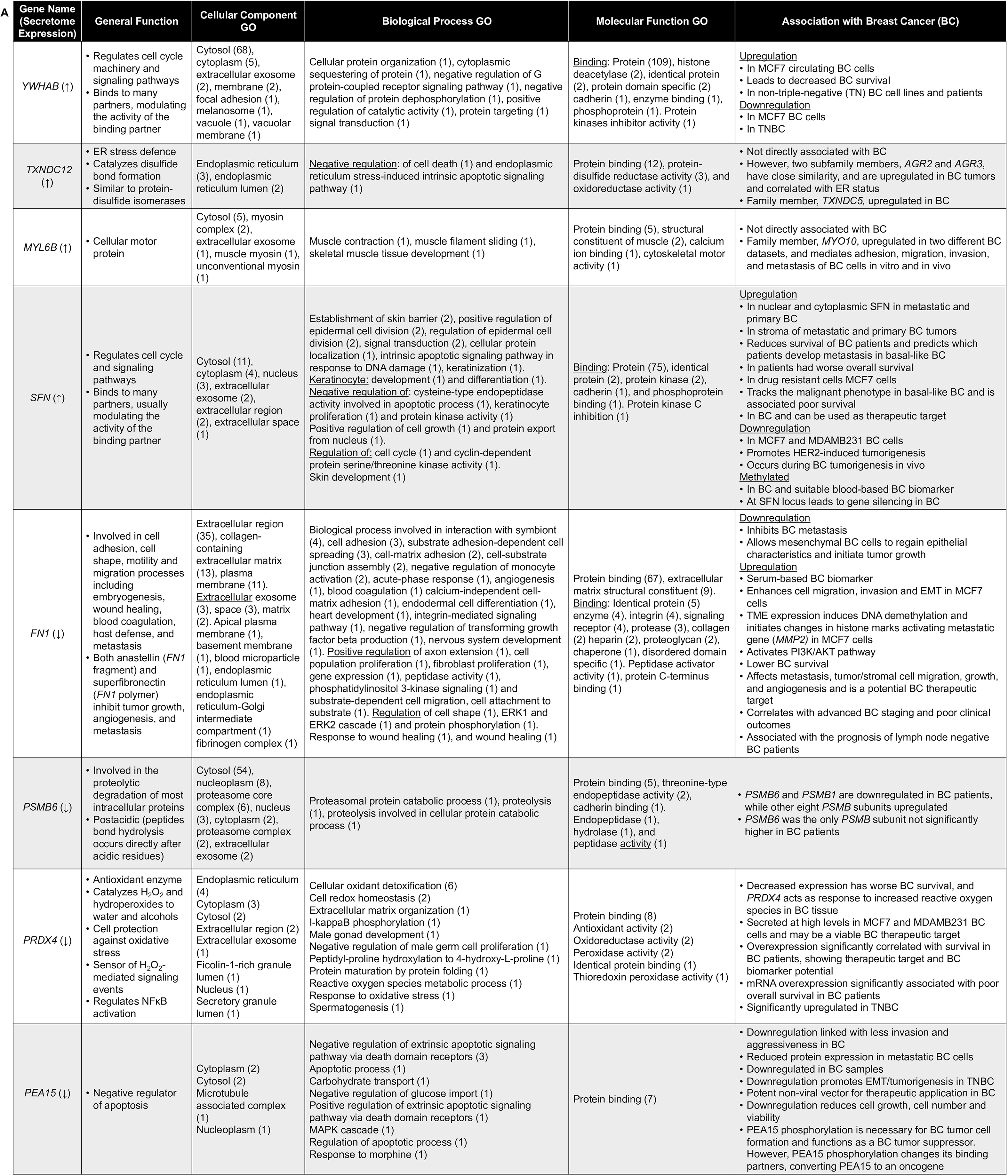

Supplement: figs5 [file mmcfigs5.jpg]

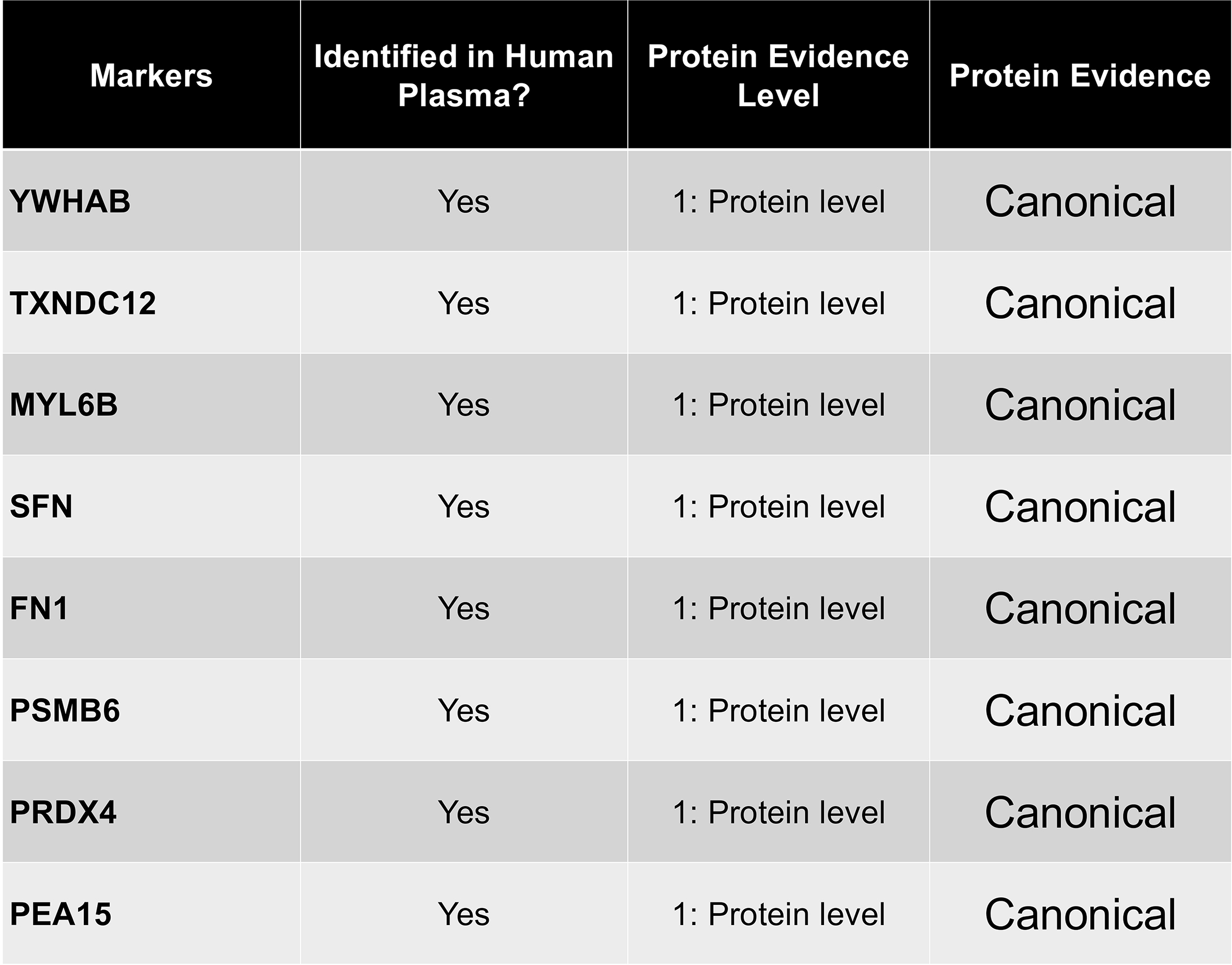

Supplement: figs6 [file mmcfigs6.jpg]
